# A Novel Probabilistic Data Association for Target Tracking in a Cluttered Environment

**DOI:** 10.3390/s16122180

**Published:** 2016-12-18

**Authors:** Xiao Chen, Yaan Li, Yuxing Li, Jing Yu, Xiaohua Li

**Affiliations:** School of Marine Science and Technology, Northwestern Polytechnical University, Xi’an 710072, China; chenxiao@mail.nwpu.edu.cn (X.C.); liyuxinglyx@sina.com (Y.L.); yujing@nwpu.edu.cn (J.Y.); lxhxy2009@163.com (X.L.)

**Keywords:** probabilistic data association (PDA), joint probabilistic data association (JPDA), interactive multi-model (IMM), combined interactive multiple model probabilistic data association (C-IMM-PDA)

## Abstract

The problem of data association for target tracking in a cluttered environment is discussed. In order to improve the real-time processing and accuracy of target tracking, based on a probabilistic data association algorithm, a novel data association algorithm using distance weighting was proposed, which can enhance the association probability of measurement originated from target, and then using a Kalman filter to estimate the target state more accurately. Thus, the tracking performance of the proposed algorithm when tracking non-maneuvering targets in a densely cluttered environment has improved, and also does better when two targets are parallel to each other, or at a small-angle crossing in a densely cluttered environment. As for maneuvering target issues, usually with an interactive multi-model framework, combined with the improved probabilistic data association method, we propose an improved algorithm using a combined interactive multiple model probabilistic data association algorithm to track a maneuvering target in a densely cluttered environment. Through Monte Carlo simulation, the results show that the proposed algorithm can be more effective and reliable for different scenarios of target tracking in a densely cluttered environment.

## 1. Introduction

As an important part of information fusion theory, target tracking has obtained more and more attention in wide applications. The main purpose of target tracking is to prevent false tracking or lost tracking, and ensure that the tracking is effective and accurate. Ship target tracking on the surface of the sea has been widely used in both military and civil fields. The performance of using radar to track surface ships are usually affected by sea clutter [1]. Due to the sea clutter, filter algorithms, such as a Kalman filter, and a series of improved filter algorithms like EKF (extended Kalman filtering) and UKF (unscented Kalman filter), are used in target tracking systems [2], and the data association is also adopted which can confirm the probability of measurement coming from the target. Previous studies have shown that the nearest-neighbor (NN) [3] algorithm works reasonably well with targets in sparse scenarios, and the probabilistic data association (PDA) [4] is suitable to track a single target in a cluttered environment, which, considering all of the measurements, falls into the validation gate. For the case of more than one target in the cluttered environment, an extension of probabilistic data association (PDA) was derived, called joint probabilistic data association (JPDA) [5], where the association probabilities are computed from the joint likelihood functions corresponding to the joint hypotheses associating all of the returns to different permutations of the targets and clutter points. Another advanced algorithm, multiple hypothesis tracking (MHT) [6], is categorized as a “deferred logic” method in which the decision about forming a new track or removing an existing track is delayed until enough measurements are collected. Recently, two popular algorithms have been derived within finite set statistics (FISST), namely, the probability hypothesis density (PHD) [7] and the cardinalized PHD (CPHD) [8] filters for multiple target tracking, which can avoid the problem of data association and can estimate both the target state and the target number directly.

Even though the JPDA has gained more and more attention in the field of target tracking, it tends to coalescence when two targets are in parallel or at a small-angle crossing. The exact nearest-neighbor probabilistic data association (ENNPDA) [9] algorithm has shown that associated events pruning may be an effective way to prevent tracking coalescence, which was found to lead to track over-commitment and an increased incidence of track divergence in the presence of clutter and absent target detections. The dramatic pruning used for ENNPDA, however, leads to an undesired sensitivity to clutter and missed detections [10]. Kennedy’s paper [11] proposed a new scaled JPDA algorithm which introduced an arbitrary positive scaling factor. When the scale factor value is infinity, the algorithm is equivalent to the ENNPDA algorithm, and when the scale factor value is one, the algorithm is equivalent to the JPDA algorithm. Thus, when the scale factor value is an appropriate intermediate value, the algorithm can not only avoid track coalescence, but can also prevent track divergence in the presence of clutter and absent target detections. How to choose a suitable scaling factor in the real tracking problem has always been the focus in multi-target tracking, and the suitable scaling factor is usually determined from the experience. To improve this, Blom has proposed a developed JPDA* [12], a coalescence-resistant version of JPDA, which performs as well as the Set JPDA [13]. Recently, a coalescence-avoiding version of JIPDA [14], called JIPDA* [15], was proposed to solve tracking coalescence. In this paper, we propose an improved version of the PDA algorithm which uses distance weighting to optimize the association probability. Then the tracking performance can do well when two targets are in parallel or at a small-angle crossing under a densely cluttered environment.

When dealing with the problem of maneuvering target tracking in the densely cluttered environment, due to the uncertainty of the target motion, the multiple model algorithm assumes that there are many possible target movement models, different movement patterns with different motion models, realizing the mutual switch between different modes. Then the maneuvering target state estimation problem is converted into a target motion pattern recognition problem. In order to achieve multiple-target motion multiple-model tracking, the interactive multiple-model (IMM) algorithm, originally developed by Blom and Bar-Shalom [16], and then the improved IMM algorithm, like AIMM [17] and VSMM [18], etc., are put forward. The application of IMM is relatively simple, and can handle complex movement patterns, which is one of the most popular algorithms used in maneuvering target tracking. The combination of PDA and IMM (IMM-PDA) [19] used in single-maneuvering target tracking was put forward. Based on IMM-PDA algorithm, the authors in [20] proposed a combined interactive multiple-model probabilistic data association (C-IMM-PDA) algorithm. Based on the frame of C-IMM-PDA, using the improved PDA filter, the performance of tracking maneuvering targets has improved.

In this paper, a novel probabilistic data association algorithm is proposed based on the distance weighting method. This algorithm not only improves the performance of single-target tracking in densely cluttered environments, but can also avoid coalescence when the targets are in parallel or in small-angle crossing scenarios. Based on the frame of C-IMM-PDA, and using the improved PDA filter, the algorithm also does well when tracking maneuvering targets in densely cluttered environments. The target tracking system is described in Section 2. In Section 3, on the basis of research of PDA, an improved probabilistic data association based on distance weighting is proposed to enhance the tracking accuracy and stability in densely cluttered environments, and also for maneuvering target tracking. Then, an improved C-IMM-PDA is presented. Simulation results showing how the performance compares to the existing algorithm are shown in Section 4. Finally, some conclusions are provided in Section 5.

## 2. Target Tracking Dynamic System in a Cluttered Environment

In a target tracking dynamic system, the major purpose is to estimate the parameters of the target that evolve sequentially with time. As measurement data become available, the unknown parameters forming a state vector are estimated sequentially using measurement data. Tracking in time with the problem of parameter estimation can be readily formalized in the following framework. In most surface ship tracking applications, the noise terms are additive, so the expression of the state equation and the measurement equation are defined as follows:

(1) $$X\left(k+1\right)=F\left(k\right)X\left(k\right)+G\left(k\right)w_{1}\left(k\right)$$

(2) $$z\left(k\right)=H(k)X(k)+w_{2}(k)$$

The state equation (Equation (1)) describes the evolution or transition of X(k) with k and assumes that the state follows a first-order Markov process. Function F(k) is a known function related with the state vector at time k−1 to the time k. Function G(k) is a known noise matrix. $w_{1}\left(k\right)$ is the process noise and its covariance is Q(k).

The measurement Equation (2) relates function z(k) to state vector X(k) through a known function H(k), $w_{2}\left(k\right)$ is the measurement noise, and its covariance is R(k). The process noise and measurement noise are independent, zero mean noise, with known covariance, respectively. In the sea clutter environment, at time k, the measurements can come from the target or the clutter, The measurements at time k are $z\left(k\right)=\left\{z_{1}\left(k\right),\cdots z_{n}\left(k\right)\right\}$. n is the measurement number at time k. The cumulative set of measurements until time k is $Z^{k}=\left\{z\left(1\right),\cdots z\left(k\right)\right\}$.

## 3. A Novel Probabilistic Data Association

### 3.1. Probabilistic Data Association (PDA) Algorithm

As for PDA, there are two assumptions: (1) a measurement can only have one source; (2) no more than one measurement can originate from a target. Suppose the set of validated measurements at time k is $z\left(k\right)={\left\{z_{i}\left(k\right)\right\}}_{i=1}^{m_{k}}$, $m_{k}$ is the validated measurements at time k. The cumulative set of measurements until time k is $Z^{k}={\left\{z\left(j\right)\right\}}_{j=1}^{k}$. The validation region is centered on the predicted measurement of the target, which is set up to accept the measurement at a certain probability. The validation region is defined as follows:

(3) $${\left[z(k)-\hat{z}(k|k-1)\right]}^{T}S^{-1}\left(k\right)\left[z(k)-\hat{z}(k|k-1)\right]\leq \gamma$$

Here, we use a Kalman filter to estimate the state of the target. The prediciton of the state and the measurement at time k are defined, respectively, as $\hat{X}(k|k-1)=F\left(k\right)\hat{X}(k-1|k-1)$ and $\hat{z}(k|k-1)=H\left(k\right)\hat{X}\left(k|k-1\right)$. $S\left(k\right)=H\left(k\right)P\left(k|k-1\right)H{\left(k\right)}^{T}+R\left(k\right)$ is the covariance matrix of innovation, $P\left(k|k-1\right)=F\left(k\right)P\left(k-1|k-1\right)F\left(k\right)+G\left(k\right)Q\left(k\right)G{\left(k\right)}^{T}$ is the covaiance of the prediction state. γ is the threshold which can be get from the chi-square distribution.

Denote association events as $\theta_{i}\left(k\right)=\left\{z_{i}\left(k\right)\;\mathrm{is}\;\mathrm{originated}\;\mathrm{from}\;\mathrm{the}\;\mathrm{target}\right\},i=1,2\cdots m_{k}$ $\theta_{i}\left(k\right)=\left\{\mathrm{none}\;\mathrm{of}\;\mathrm{the}\;\mathrm{measurement}\;\mathrm{is}\;\mathrm{originated}\;\mathrm{from}\;\mathrm{the}\;\mathrm{target}\right\},i=0$. The probability of either the measurements originating from the target or clutter (false alarm) is expressed as the likelihood (Equation (4)) condition that all of the measurements lie in the validation region:

(4) $$\beta_{i}\left(k\right)=P\left\{\theta_{i}\left(k\right)|Z^{k}\right\}$$

As we know, the definition of events are mutually exclusive and limited: $\sum_{i=0}^{m_{k}}\beta_{i}\left(k\right)=1$. Here we use a Kalman filter to estimate the target state. The expression is shown as follows:

(5) $$\hat{X}\left(k|k\right)=\sum_{i=0}^{m_{k}}\beta_{i}\left(k\right){\hat{X}}_{i}\left(k|k\right)=\hat{X}\left(k|k-1\right)+K\left(k\right)\left(\sum_{i=1}^{m_{k}}\beta_{i}\left(k\right)v_{i}\left(k\right)\right)$$

If there is no measurement that falls into the validation region, namely i=0, we use the state prediction to approximate the updated values of the target state. ${\hat{X}}_{0}\left(k\right)=\hat{X}\left(k|k-1\right)$. $v_{i}\left(k\right)=Z_{i}\left(k\right)-H\left(k\right)\hat{X}\left(k|k-1\right)$ is the innovation, $v\left(k\right)=\sum_{i=1}^{m_{k}}\beta_{i}\left(k\right)v_{i}\left(k\right)$ is the combined innovation, and the Kalman gain is $K\left(k\right)=P\left(k|k-1\right)H^{T}\left(k\right)S^{-1}\left(k\right)$.

The error covariance associated with the updated state estimation can be expressed as follows:

(6) $$P\left(k|k\right)=P\left(k|k-1\right)\beta_{0}\left(k\right)+\left[1-\beta_{0}\left(k\right)\right]P^{c}\left(k|k\right)+\tilde{P}\left(k\right)\;P^{c}\left(k|k\right)=\left[I-K\left(k\right)H\left(k\right)\right]P\left(k|k-1\right)\;\tilde{P}\left(k\right)=K\left(k\right)\left[\sum_{i=1}^{m_{k}}\beta_{i}\left(k\right)v_{i}\left(k\right){v_{i}}^{T}\left(k\right)-v\left(k\right)v^{T}\left(k\right)\right]K^{T}\left(k\right)$$

### 3.2. A New Association Probability of PDA

The association probability is the likelihood of $\theta_{i}\left(k\right)$ on the condition that all of the measurement lie in the validation region as explained by Equation (3). Using the parametric PDA [4], the association probability is as follows:

(7) $$\beta_{i}\left(k\right)=\{\begin{array}{cc} e_{i}/\left(b+\sum_{j=1}^{m_{k}}e_{j}\right) & i=1,\ldots m_{k}\\ b/\left(b+\sum_{j=1}^{m_{k}}e_{j}\right) & i=0 \end{array}$$

where $e_{i}≜\exp\left(-\frac{1}{2}{v_{i}}^{T}\left(k\right)S^{-1}\left(k\right)v_{i}\left(k\right)\right)$; $b≜\lambda {\left|2\pi S\left(k\right)\right|}^{\frac{1}{2}}\left(1-P_{d}P_{g}/P_{d}\right)$, $P_{d}$ is the probability of detection, and $P_{g}$ is the probability that the target measurement falls into the validation region.

$\beta_{i}\left(k\right)$ is an important parameter which directly influences the estimation of the target state. The PDA algorithm considers all of the measurements falling into the validation region, and there is no clear and effective relationship between the validation measurement and the prediction measurement. Since the clutter is distributed randomly in position, number, and density in the tracking space, the measurement originating from the target is more likely near the predicted measurement. Thus, this important prior information is introduced as a distance weight to optimize the association probability. The weight calculation method in the literature [21] has certain limitations, namely, when the number of validation measurements is just one, the value will be infinite, which can lead to filter failure. Here we propose a new expression of distance weight:

(8) $$\omega_{i}\left(k\right)=\frac{1/d_{i}\left(k\right)}{\sum_{j=1}^{m_{k}}1/d_{i}\left(k\right)}$$

where $d_{i}\left(k\right)$ is the Mahalanobis distance between measurement i and the prediction measurement at time k, the expression is $d_{i}\left(k\right)=\left(z_{i}\left(k\right)-{\hat{z}}_{i}\left(k|k-1\right)\right)S\left(k\right){\left(z_{i}\left(k\right)-{\hat{z}}_{i}\left(k|k-1\right)\right)}^{T}$. $z_{i}\left(k\right)$ is the validated measurement at time k, ${\hat{z}}_{i}\left(k|k-1\right)$ is the prediction measurement, and the expression is ${\hat{z}}_{i}\left(k|k-1\right)=H\left(k\right)\hat{X}\left(k|k-1\right)$. S(k) is the covariance matrix of innovation in the Kalman filter, and its expression is $S\left(k\right)=H\left(k\right)P\left(k|k-1\right)H{\left(k\right)}^{T}+R\left(k\right)$.

The association probability expression with the distance weighting is show below:

(9) $$\beta_{i}\left(k\right)=\beta_{i}\left(k\right)\omega_{i}\left(k\right)\;i=1,2\cdots m_{k}$$

The normalized association probability expression is show as Equation (10):

(10) $$\beta_{i}\left(k\right)=\beta_{i}\left(k\right)/\sum_{j=0}^{m_{k}}\beta_{j}\left(k\right)\;i=0,1,\cdots m_{k}$$

Here, with the new association probability, we use a Kalman filter to estimate the target state. The calculation is shown in Equations (5) and (6).

### 3.3. Improved C-IMM-PDA Algorithm

The traditional algorithm is just a combination of the interactive multiple model and the probabilistic data association together. On the one hand, each model uses an independent validation region to obtain the validated measurement. However, for the entire system, the validation region may not be optimal, which can increase the target estimation error, and finally lead to a lost target or tracking the wrong target. On the other hand, with the increase in the number of targets and the number of target motion modes, the computational cost will also increase. Thus, the literature [20] proposes a new IMM-PDA structure (combined interactive multiple-model probabilistic data association algorithm, C-IMM-PDA), which uses the same validation region to obtain the validated measurement, and also uses comprehensive measurement to update and estimate the target state. This method can better realize target tracking and save on the computational cost. Here, based on C-IMM-PDA, we use the improved PDA filter to obtain the association probability which can enhance the accuracy of the estimation target state. The improved C-IMM-PDA algorithm implementation stated is as follows.

1. Interaction Mixed with the Estimate from the Previous Time

Starting with ${\hat{X}}^{j}\left(k-1|k-1\right)$, its covariance, $P^{j}\left(k-1|k-1\right)$ and the model probability $u^{j}\left(k-1\right)$, the mixed initial condition for the filter matched to model j can be expressed as follows:

(11) $$\begin{array}{ll} {\hat{X}}^{0j}\left(k-1|k-1\right) & =\sum_{j=1}^{M}{\hat{X}}^{j}\left(k-1|k-1\right)u_{i|j}\left(k-1|k-1\right)\\ P^{0j}\left(k-1|k-1\right) & =\sum_{j=1}^{M}u_{i|j}\left(k-1|k-1\right)\{P^{j}\left(k-1|k-1\right)+\cdots \\ & \left[{\hat{X}}^{j}\left(k-1|k-1\right)-{\hat{X}}^{0j}\left(k-1|k-1\right)\right]{\left[{\hat{X}}^{j}\left(k-1|k-1\right)-{\hat{X}}^{0j}\left(k-1|k-1\right)\right]}^{T}\} \end{array}$$

where $u_{i|j}\left(k-1|k-1\right)$ is the mixed probability, $u_{i|j}\left(k-1|k-1\right)=p_{ij}u^{j}\left(k-1\right)/c_{j}$, $c_{j}$ denotes the normalizing factor, and $c_{j}=\sum_{j=1}^{M}p_{ij}u^{j}\left(k-1\right)$. $p_{ij}$ is the switch probability from model i to model j.

2. Submodel Filter and Prediction

With the mixed initial state ${\hat{X}}^{0j}\left(k-1|k-1\right)$ and its covariance $P^{0j}\left(k-1|k-1\right)$, here we use a Kalman filter to estimate the target state, the predicted state ${\hat{X}}^{j}\left(k|k-1\right)$, and its corresponding covariance $P^{j}\left(k|k-1\right)$, the predicted measurement ${\hat{z}}^{j}\left(k\right)$, and the covariance matrix of innovation $S^{j}\left(k\right)$ for each motion model j can be expressed as follows:

(12) $$\begin{array}{l} {\hat{X}}^{j}\left(k|k-1\right)=F^{j}{\hat{X}}^{0j}\left(k-1|k-1\right)\\ P^{j}\left(k|k-1\right)=F^{j}P^{0j}\left(k-1|k-1\right){\left(F^{j}\right)}^{T}+Q^{j}\left(k-1\right)\\ {\hat{z}}^{j}\left(k\right)=H{\hat{X}}^{j}\left(k|k-1\right)\\ S^{j}\left(k\right)=HP^{j}\left(k|k-1\right)H^{T}+R^{j}\left(k\right)\begin{array}{cc} & j=1,2\cdots M \end{array} \end{array}$$

Here, we use the comprehensive prediction of the measurement and the comprehensive prediction covariance matrix tectonic tracking region. Its definitions are as follows:

(13) $$\hat{z}\left(k\right)=\hat{z}\left(k|k-1\right)=E\left[z\left(k\right)|z^{k-1}\right]$$

(14) $$S\left(k\right)=S\left(k|k-1\right)=E\left\{\left[z\left(k\right)-\hat{z}\left(k\right)\right]\cdot {\left[z\left(k\right)-\hat{z}\left(k\right)\right]}^{T}|Z^{k-1}\right\}$$

Here, using definitions from Equations (13) and (14), the parameters of the comprehensive validation region can be given as:

(15) $$\begin{array}{l} \hat{z}\left(k\right)=\sum_{j=1}^{M}u^{j}\left(k\right){\hat{z}}^{j}\left(k\right)\\ S\left(k\right)=\sum_{j=1}^{M}u^{j}\left(k\right)\left\{S^{j}\left(k\right)+\left[z\left(k\right)-\hat{z}\left(k\right)\right]\cdot {\left[z\left(k\right)-\hat{z}\left(k\right)\right]}^{T}\right\} \end{array}$$

Set $d^{2}\left(k\right)=\left[z(k)-\hat{z}(k)\right]S^{-1}\left(k\right){\left[z(k)-\hat{z}(k)\right]}^{T}$. Assuming $\left[z(k)-\hat{z}(k)\right]$ obeys the normal distribution so that $d^{2}\left(k\right)$ obeys the chi-square distribution with the degree of freedom $n_{z}$, we use the method of hypothesis testing to determine whether z(k) falls into the validation region.

Suppose $m_{k}$ is the number of validated measurements at time k. Each filter using comprehensive measurement as the prediction measurement to update the state, the update equations are the same as the normal Kalman filter. Comprehensive measurement can be expressed as follows:

(16) $${\bar{z}}^{j}\left(k\right)=E\left[z\left(k\right)\right]=\sum_{l=0}^{m_{k}}E\left[z\left(k\right)|\theta_{l}^{j}\left(k\right)\right]\beta_{l}^{j}\left(k\right)=\sum_{l=0}^{m_{k}}z_{l}^{j}\beta_{l}^{j}\left(k\right)$$

where $\beta_{l}^{j}\left(k\right)$ can be obtained from Equation (10).

Using Equation (16) to update each filter will lead to the estimation covariance being large; here we use Equation (17) to correct its covariance:

(17) $$P_{i}^{j}\left(k|k\right)=P_{i}^{j}\left(k|k-1\right)\beta_{0}^{j}\left(k\right)+\left[1-\beta_{0}^{j}\left(k\right)\right]P_{i}^{c}\left(k|k\right)+{\tilde{P}}_{i}\left(k\right)\;{P_{i}}^{c}\left(k|k\right)=\left[I-K_{i}\left(k\right)H_{i}\left(k\right)\right]P_{i}^{j}\left(k|k-1\right)\;\begin{array}{ll} {\tilde{P}}_{i}\left(k\right) & =K_{i}\left(k\right)\{\beta_{0}^{j}\left(k\right)\left[{\tilde{z}}_{i}\left(k\right){\tilde{z}}_{i}\left(k\right)-\bar{v}\left(k\right){\bar{v}}^{T}\left(k\right)\right]+\cdots \\ & \sum_{l=1}^{m_{k}}\beta_{l}^{j}\left(k\right)v_{l}\left(k\right)v_{l}^{T}\left(k\right)-\bar{v}\left(k\right){\bar{v}}^{T}\left(k\right)\}K_{i}^{T}\left(k\right) \end{array}$$

3. Model Probability Update

The model probability is updated by $u_{l}\left(k\right)$ as in Equation (18):

(18) $$u_{j}\left(k\right)=\frac{L_{j}\left(k\right)\sum_{i=1}^{M}u_{i}\left(k-1\right)p_{ij}}{\sum_{j=1}^{M}L_{j}\left(k\right)\sum_{i=1}^{M}u_{i}\left(k-1\right)p_{ij}}$$

Here, considering model probabilities update as an independent part of IMM, $L_{j}\left(k\right)=\frac{\exp\left(-\frac{1}{2}{\tilde{z}}^{j}\left(k\right)S^{j}\left(k\right){\left({\tilde{z}}^{j}\right)}^{T}\left(k\right)\right)}{\sqrt{\left|2\pi S^{j}\left(k\right)\right|}}$ where ${\tilde{z}}^{j}\left(k\right)={\bar{z}}^{j}\left(k\right)-{\hat{z}}^{j}\left(k\right)$.

4. Interaction Ouput Estimation Results

Finally, the model-conditioned estimates are calculate using Equation (19).

(19) $$\begin{array}{l} \hat{X}\left(k\right)=\sum_{j=1}^{M}u_{j}\left(k\right){\hat{X}}_{j}\left(k|k\right)\\ P\left(k\right)=\sum_{j=1}^{M}u_{j}\left(k\right)\left\{P_{j}\left(k|k\right)+\left[X_{j}\left(k|k\right)-\hat{X}\left(k\right)\right]{\left[X_{j}\left(k|k\right)-\hat{X}\left(k\right)\right]}^{T}\right\} \end{array}$$

## 4. Simulation and Analysis

### 4.1. The Dynamic Model

Assume the target state is $X(k)=[x,\dot{x},y,\dot{y}]$. The two-dimensional plane dynamic model of the target and the measurement equation can be described by the following equations:

(20) $$\begin{array}{l} X(k+1)=F\left(k\right)X(k)+G\left(k\right)w_{1}\left(k\right)\\ z\left(k\right)=H\left(k\right)X(k)+w_{2}(k) \end{array}$$

In the constant velocity model (CV model), the movement characteristic is the velocity of the target, which remains the same, and the model parameters are as follows: where $F(k)=\left[\begin{array}{cccc} 1 & T & 0 & 0\\ 0 & 1 & 0 & 0\\ 0 & 0 & 1 & T\\ 0 & 0 & 0 & 1 \end{array}\right]$ G=[T220T00T220T] $H=\left[\begin{array}{cccc} \begin{array}{c} 1\\ 0 \end{array} & \begin{array}{c} 0\\ 0 \end{array} & \begin{array}{c} 0\\ 1 \end{array} & \begin{array}{c} 0\\ 0 \end{array} \end{array}\right]$.

The turning motion of the target is usually referred to as a coordinated turn (CT model). With a known target turning rate ω, the model parameters are as follows: where $F(k)=\left[\begin{array}{cccc} 1 & \frac{\sin\omega T}{\omega } & 0 & \frac{\cos\omega T-1}{\omega }\\ 0 & \cos\omega T & 0 & -\sin\omega T\\ 0 & \frac{1-\cos\omega T}{\omega } & 0 & \frac{\sin\omega T}{\omega }\\ 0 & \sin\omega T & 0 & \cos\omega T \end{array}\right]$ $G=\left[\begin{array}{c} \begin{array}{cc} \frac{T^{2}}{2} & 0 \end{array}\\ \begin{array}{cc} T & 0 \end{array}\\ \begin{array}{cc} 0 & \frac{T^{2}}{2} \end{array}\\ \begin{array}{cc} 0 & T \end{array} \end{array}\right]$ $H=\left[\begin{array}{cccc} \begin{array}{c} 1\\ 0 \end{array} & \begin{array}{c} 0\\ 0 \end{array} & \begin{array}{c} 0\\ 1 \end{array} & \begin{array}{c} 0\\ 0 \end{array} \end{array}\right]$.

### 4.2. Simulation and Analysis of Non-Maneuvering Target Tracking

In this section, the performance of the improved PDA algorithm are evaluated and compared with the existing method of PDA. Assume detection probability $P_{d}=0.9$, the clutter density $\lambda =1/{\mathrm{km}}^{2}$. The gate threshold is set to γ=9, which corresponds to a two-dimensional gating probability of $P_{g}=0.989$, sampling interval T=1 s, the process noise variance $q_{cv}=0.05\;m\;$, and the measurement noise variance r=100 m . In order to compare the performance, 100 Monte Carlo simulations have been performed.

#### 4.2.1. Simulation 1: Single Target Tracking Simulation Scenario

**Figure 1:**
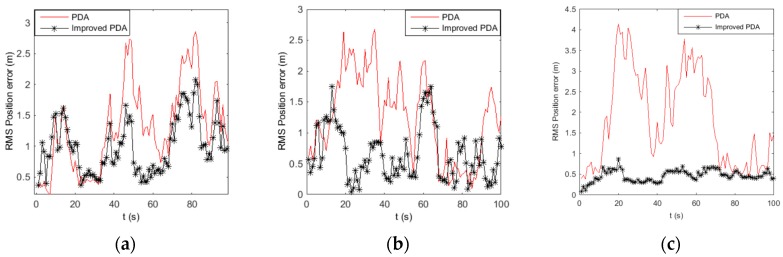
RMS position error statistics using PDA and improved PDA in different density clutter. (**a**) λ=1; (**b**) λ=10; and (**c**) λ=50.

The initial state is x=[1000 m 10 m/s 1000 m −5 m/s]. The target stays at a constant velocity between 0 s and 100 s. The root mean square (RMS) position error of the target using different methods in different clutter densities are show in Figure 1.

Figure 1 shows the RMS position errors, which is computed with clutter densities of 1, 10, and 50, respectively. From Figure 1a, we can see the two algorithms exhibit similar RMS position error when clutter density is λ=1, while when the clutter density increase, the RMS position error of the improved PDA is smaller than the PDA, especially when clutter density λ=50, which can be seen from Figure 1c. In the improved PDA algorithm, we use the new association probability to realize the data association which can optimize the probability of the measurement originating from the target, so the improved algorithm can perform better to estimate the target state accurately. From the above, we know that improved PDA can improve the tracking performance in densely cluttered environments when tracking a single non-maneuvering target.

#### 4.2.2. Simulation 2: Two-Target Tracking Simulation Scenario

(1) Two parallel-target tracking. The initial states are $x_{a}=\left[200\;m\;15\;m/s\;100\;m\;10\;m/s\right]$, $x_{b}=\left[200m\;15m/s\;300m\;10m/s\right]$, the target stays at a constant velocity between 0 s and 50 s.
(2) Two small-angle crossing target tracking. The initial states are $x_{a}=\left[100\;m\;20\;m/s\;300\;m\;0\;m/s\right]$, $x_{b}=\left[100m\;20m/s\;200m\;5m/s\right]$, the target stays at a constant velocity between 0 s and 100 s.

**Figure 2:**
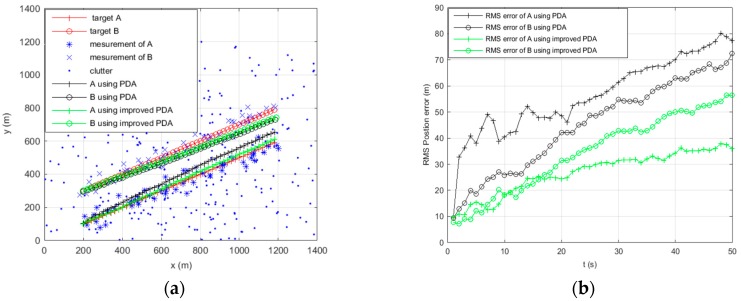
Tracking two parallel targets using PDA and improved PDA when clutter λ=1. (**a**) Tracking of the target position; and (**b**) RMS position error of the target.

**Figure 3:**
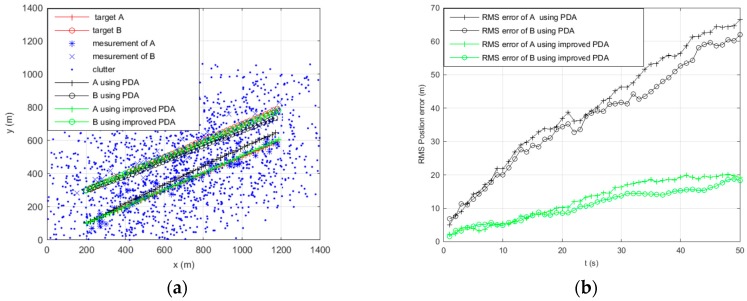
Tracking two parallel targets using PDA and improved PDA when clutter λ=5. (**a**) Tracking of the target position; and (**b**) RMS position error of the target.

**Figure 4:**
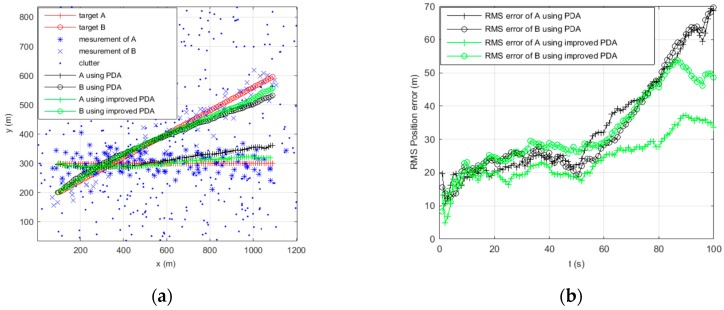
Tracking two crossing targets using PDA and improved PDA when clutter λ=1. (**a**) Tracking of the target position; and (**b**) RMS position error of the target.

**Figure 5:**
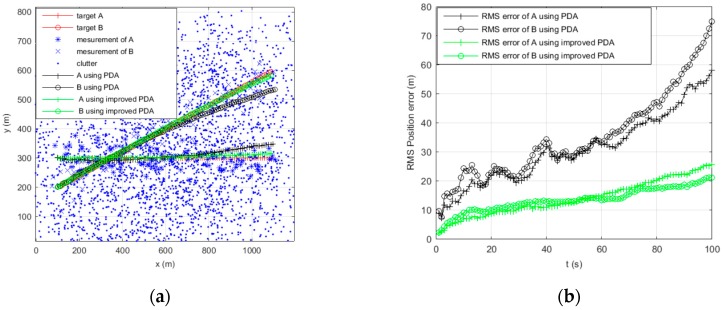
Tracking two crossing targets using PDA and improved PDA when clutter λ=5. (**a**) Tracking of the target position; and (**b**) RMS position error of the target.

Figure 2, Figure 3, Figure 4 and Figure 5 show the trajectories of two targets and the RMS position error statistics using PDA and improved PDA in different scenarios. As for two parallel targets tracking, with the increase of the clutter density, Figure 2b and Figure 3b show that the RMS position of the improved PDA algorithm is always smaller than the PDA algorithm. Figure 2a and Figure 3a show that the improved PDA can be more accurate to realize target tracking. As for two crossing targets tracking, we can obtain similar results from Figure 4 and Figure 5. From the above simulation results, we can see that the improved PDA algorithm performs better than the PDA algorithm whenever the two targets move in parallel or cross in the densely cluttered environment. This can be attributed to the reduction of the association probability of false measurement and, at the same time, enhance the association probability originating from the target. The improved algorithm can estimate the target state more accurately to avoid track coalescence whatever two targets are in parallel or at a small-angle crossing in a densely cluttered environment.

### 4.3. Simulation and Analysis of Maneuvering Target Tracking

**Figure 6:**
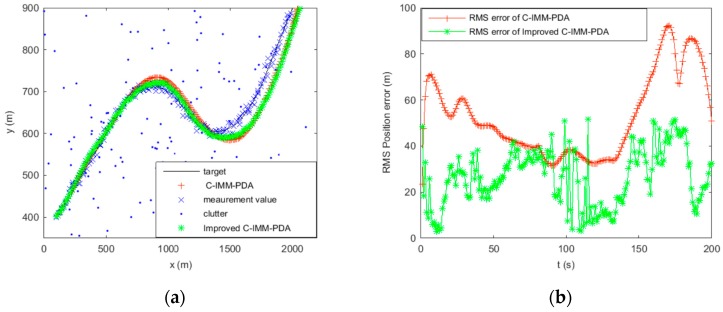
Maneuvering target tracking using C-IMM-PDA and improved C-IMM-PDA when clutter λ=1. (**a**) Tracking of the target position; and (**b**) RMS position error of the target.

**Figure 7:**
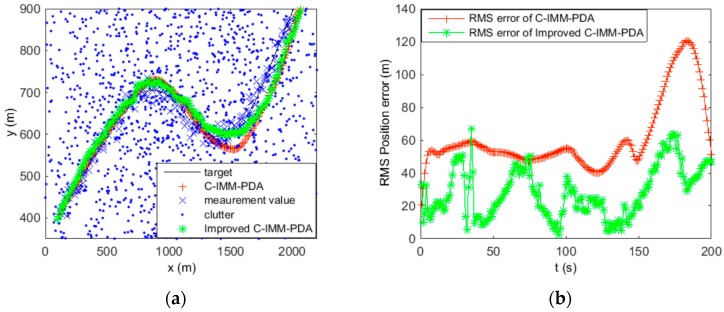
Maneuvering target tracking using C-IMM-PDA and improved C-IMM-PDA when clutter λ=10. (**a**) Tracking of the target position; and (**b**) RMS position error of the target.

In this section, the performance of the proposed algorithm is evaluated and compared with the existing method of C-IMM-PDA. The initial states x=[1000 m 10 m/s 400 m 5 m/s], the target initially stays at a constant velocity between 0 s and 50 s, with a coordinated turn ω= −1 rad/s between 50 s and 100 s, then with a coordinated turn ω= 1 rad/s between 100 s and 150 s and, finally, a straight line with constant velocity between 150 s and 200 s. Assuming the detection probability $P_{d}=0.9$, the clutter measurement density $\lambda =1/{\mathrm{km}}^{2}$. The gate threshold is set to γ=16 which corresponds to a two-dimensional gating probability of $P_{g}=0.9997$. Sampling interval T=1 s, the process noise variance for CV model $q_{cv}=0.05\;m\;$, the CT model $q_{ct}=0.015\;m\;$, and the measurement noise variance r=100 m . The transition probability matrix of IMM models is $P_{ij}=\left[\begin{array}{ccc} 0.9 & 0.05 & 0.05\\ 0.05 & 0.9 & 0.05\\ 0.05 & 0.05 & 0.9 \end{array}\right]$, $u_{0}=[\begin{array}{ccc} 0.5 & 0.2 & 0.3 \end{array}]$. In order to compare the performance, 50 Monte Carlo simulations have been performed. The simulation results are shown in Figure 6 and Figure 7.

Figure 6 and Figure 7 show the trajectories of a single maneuvering target and the RMS position error statistics using C-IMM-PDA and improved C-IMM-PDA with clutter densities of 1 and 10, respectively. As it would be expected, along with the increase of clutter density, the RMS position error shows the two algorithms exhibiting a decrease in performance. However, the degradation in performance is less for the improved C-IMM-PDA algorithm than for the C-IMM-PDA algorithm. In the improved C-IMM-PDA algorithm, using the same validated region can obtain the effective validated region for the whole system, and using the improved PDA algorithm can make the estimation of target more accurate. Additionally, when using the same validated region, this algorithm can reduce the computational cost. Simulation results show that the improved C-IMM-PDAF algorithm can be more effective to achieve high reliability in target tracking in densely cluttered environments.

## 5. Conclusions

In this paper, we proposed a novel probabilistic data association algorithm based on distance weighting for target tracking. Due to clutter distribution being random, and the measurement originating from the target is more likely closer to the predicted measurement, this important prior information is introduced as a distance weight to optimize the association probability, which can enhance the association probability of the measurement originating from the target. When the improved algorithm is applied to non-maneuvering target tracking, the algorithm can be more accurate to realize the single non-maneuvering target tracking in a densely cluttered environment. For two-target tracking, it can avoid tracking coalescence whatever two targets are in parallel or at a small-angle crossing in a densely cluttered environment. For maneuvering target tracking in densely cluttered environments, based on the frame of C-IMM-PDA, we use the improved PDA to realize the data association and a Kalman filter to estimate the target state. Simulation results show the performance of the improved C-IMM-PDA algorithm is better than the C-IMM-PDA when tracking maneuvering targets in densely cluttered environments. Above all, with the increase of clutter density, the performance of the improved algorithm did not degrade significantly, which can ensure the real-time processing and accuracy of target tracking in densely cluttered environments.
